# Health and Environmental Effect of Advanced Materials and Fine Particles

**DOI:** 10.3390/biom12111579

**Published:** 2022-10-28

**Authors:** Il Je Yu

**Affiliations:** 1 HCT America, Morgan Hill, CA 95131, USA; u1670916@chollian.net; 2 HCT, 74, Seoicheon-ro 578beon-gil Majang-myeon, Icheon-si 17383, Korea

With the recent development in material sciences, advanced materials have become terms widely used for new materials. Advanced materials comprise biopolymers, active materials, hybrids, composites, nanomaterials, structural materials, particle systems, advanced fibers, metamaterials, and advanced manufacturing. EU also defines advanced materials as “any material that, through the precise control of its composition and internal structure, features a series of exceptional properties (mechanical, electric, optic, magnetic, etc.) or functionalities (self-repairing, shape change, decontamination, transformation of energy, etc.) that differentiate it from the rest of the universe of materials; or one that, when transformed through advanced manufacturing techniques, features these properties or functionalities”. OECD WPMN and OECD Biotechnology are also interested in the safety and risk assessment of advanced materials, and several activities regarding advanced materials are ongoing. In addition, fine particles, including ultrafine particles, are gradually garnering attention for the human health effect. Particle sizes, in particular those that are less than 2.5 microns (fine) or 100 nm (ultrafine), have been known to affect human health. Similar to advanced materials, their specific properties, such as high number concentration, large surface area, and their tendency to penetrate and translocate to the blood system and other organs, present a risk to human health. 

The scopes of this Special Issue include health effects and toxicity (in vivo and in vitro) of advanced materials and fine particles, toxicokinetics and pharmacokinetics of advanced materials and fine particles, environmental toxicity of advanced materials and fine particles, exposure assessment in workplaces producing or handling advanced materials and fine particles, risk assessment of advanced materials and fine particles, and policies regarding the safe management of advanced materials and fine particles.

After an indium-related lung disease was reported in a display manufacturing factory in Korea, Kim et al. [1] conducted an assessment of occupational exposure at an indium tin oxide (ITO) powder manufacturing plant. They found that the respirable indium concentration exceeded the recently set ACGIH TLV for the respirable fraction of indium dust. The concentration of indium NPs was between 0.003 and 0.010 × 10^−2^ mg/m^3^, accounting for only 0.4% of the total and 2.7% of the respirable indium particles. They suggested that follow-up studies need to be conducted on other ITO sputtering target polishing and milling processes, which typically generate more airborne nanoparticles, to further investigate the effects of indium on workers and to develop indium exposure mitigation technologies

Lee et al. [2] investigated the age and gender effects of diesel exhaust particles (DEP) exposure on genotoxicity in C57BL/6 mice. They found that DEP exposure was not associated with an increase in the frequency of micronucleated polychromatic erythrocytes and did not induce systemic genotoxic effects in the bone marrow without an age effect but induced a significant increase in DNA damage in DEP-exposed mouse lung specimens in the comet assay with age. This finding suggested that the age factor should be considered to better understand the cellular adverse effects of PM2.5.

Based on epidemiological evidence indicating DEP’s association with an increase in the relative risk of lung cancer, Lee et al. [3] attempted to study the genotoxic effects of DEP at the chromosomal level using normal embryonic human lung fibroblast cells with micronuclei (MN) and comet assays. They found that DEP exposure induced DNA damage at the chromosomal level in normal human lung cells and provided information on the expression of genes associated with genotoxic stress.

DEP have been known to adversely affect the respiratory system and to exacerbate lung diseases, resulting in high mortality rates. Kim et al. [4] studied the effects of DEP pre-exposure on lipopolysaccharide-induced acute lung injury and identified the roles of interleukin (IL)-17 in mice. They found that re-exposure to DEP synergistically exacerbated pulmonary acute lung inflammation and granulomatous inflammation/pulmonary fibrosis, upregulated IL-17 cytokine-mediated collagen I and TGF-β1, and activated partially LPS-induced NLRP3 inflammasome signaling.

The immunotoxicity of silver nanoparticles (AgNPs) to neutrophils has been known to be caused by reactive oxygen species (ROS). The immune reactions of neutrophils include the expulsion of webs of DNA surrounded by histones and granular proteins. These webs of DNA are termed neutrophil extracellular traps (NETs). NETs allow neutrophils to catch and destroy pathogens in extracellular spaces. Kang et al. [5] investigated how AgNPs stimulate neutrophils, specifically focusing on NETs. They found that AgNP induced NETs through ROS, peptidyl arginine deiminase, and neutrophil elastase.

Jang et al. [6] studied the size effect of AgNPs (5 nm and 100 nm) on the endothelial and epithelial cells by assessing gene expression with microarray. Their results showed that 5 nm AgNP, but not 100 nm AgNP at a high concentration, induced cytotoxicity and that 5 nm AgNP exposure increased interleukin (IL)-8 and IL-11 gene expression in the early stages, with noticeable variation in the expression of oxidative stress-related genes such as cell death-, apoptosis-, and cell survival-related genes in the early stages.

Silver has been used as a thermal interphase material (TIM) due to its high intrinsic thermal conductivity, but it is expensive and toxic. Thus, the possible use of copper as TIM, an earth-abundant element and essential micronutrient for humans, was tested for TIM. Kim et al. [7] synthesized a copper-based multi-dimensional filler composed of three-dimensional microscale copper flakes, one-dimensional multi-walled carbon nanotubes (MWCNTs), and zero-dimensional copper nanoparticles treated in a microwave oven to create a safe and low-cost TIM with high thermal conductivity. They found that copper-based TIM could be an industrially useful heat-dissipating material with high thermal conductivity and low cost.

Finally, Esposito et al. [8] presented the limitation of LCAs (life cycle assessments) in investigating engineered nanomaterials’ (ENMs) potential environmental impact. Because LCA focused on the environmental impact of the production phase, information on their environmental impact is lacking due to their in situ employment. They illustrated the development of an eco-design framework and reviewed the application of ecotoxicology as a valuable strategy for developing eco-safe engineered nanomaterials (ENM) for environmental remediation. Moreover, we described the currently available ENMs for marine environment remediation and discussed their pros and cons in safe environmental applications together with the need to balance benefits and risks promoting an environmentally safe nano-remediation (eco-safe) for the future.

## Data Availability

Not applicable.
